# Mosaic ageing: from organ-specific decline to the cause of death

**DOI:** 10.1098/rstb.2025.0335

**Published:** 2026-11-12

**Authors:** Pablo Burraco, Jelle Boonekamp

**Affiliations:** ^1^ Department of Ecology and Evolution, Doñana Biological Station, Spanish National Research Council (CSIC), Seville, Spain; ^2^ School of Biodiversity, One Health & Veterinary Medicine, University of Glasgow, Glasgow, UK

**Keywords:** biological age, exposome, frailty, functional modularity, organ networks, ageing biomarkers, heterogeneous ageing

## Abstract

Ageing is often framed as a systemic, uniform process. However, growing evidence shows that ageing occurs unevenly across cell types, tissues and organs, generating heterogeneous within-individual trajectories. This mosaic perspective highlights the myriad cross-tissue ageing patterns that emerge from interactions among development, the genome and environmental and lifestyle exposures. The way these uneven trajectories play out may determine the emergence of specific ageing-related diseases and ultimately the cause of death. Here, we combine insights from molecular biology, biogerontology and evolutionary ecophysiology to develop an integrative view of mosaic ageing. We first describe the proximate mechanisms underlying organ-specific ageing variation. These mechanisms are moulded through age-specific natural selection, embodying trade-offs between maintenance, function and fitness. We then review tools for quantifying organ-specific ageing, ranging from classical measures such as telomere dynamics to high-resolution approaches including epigenetic, proteomic and multi-omic clocks, single-cell atlases and longitudinal imaging. Finally, we discuss how combining these tools with causal inference methods can help identify drivers of context- and species-dependent senescence and life-history variation and enable organ-targeted biomedical interventions. Overall, this perspective calls for moving beyond systemic models of ageing towards an integrative framework that places heterogeneous organ-specific processes at the centre of ageing research.

This article is part of the theme issue ‘Developmental circuits in ageing’.

## 1. Introduction: the elusive concept of ageing—is it uniform?

Although ageing is a near-universal feature of living organisms, it remains one of biology's most intriguing and least understood phenomena [1–3]. Depending on the context, ageing has been defined as the occurrence of diseases, the accumulation of by-products of metabolism from developmental and environmental influences or, not mutually exclusive, the outcome of the balance between growth, somatic maintenance and reproductive effort [4–7]. Moreover, ageing has often been regarded as a uniform and systemic process, a gradual organism-wide deterioration governed by a shared set of molecular pathways [3,8]. This systemic view has driven the search for the so-called hallmarks of ageing and has heavily influenced our thinking about a potential cure against ageing [8–10]. However, a growing body of biomedical and evolutionary studies increasingly questions the notion of uniform ageing: rather than a whole-body (single) trajectory, ageing appears to occur unevenly across organs, tissues and even cell types [11–14]. Here, we use the term *mosaic ageing* to denote this patterned, within-individual heterogeneity in ageing trajectories across biological scales, encompassing differences in onset, rate and inter-organ or tissue interactions [14,15]. Other terms used to describe within-body ageing heterogeneity are outlined in box 1.

**Box 1. Terminological landscape of within-body ageing heterogeneity**

Several terms have been used to describe uneven ageing across organs and/or tissues. Some of these words have occasionally been used interchangeably, but we take them to have slightly different inherent meanings.

**Heterogeneous ageing** or **differential ageing** broadly describe variability in ageing trajectories. When applied in context to describe variation across organs and tissues, these words do not necessarily imply an organizational or mechanistic framework [13,16].

**Asynchronous ageing** refers primarily to differences in the timing of decline among tissues, organs or phenotypic traits, but it does not necessarily encompass differences in the rate, pattern or form of ageing [17].

**Modular ageing** draws on the concept of functional modules existing within the body and may imply partial independence of senescence among body parts [18].

**Segmental ageing** typically refers to anatomical or pathological restriction of ageing-like phenotypes to specific tissue degeneration, as observed in progeroid syndromes [19].

**Ageotypes** describe individual-specific ageing patterns, primarily capturing between-individual differences rather than within-body organ heterogeneity [20].

**Mosaic ageing**, *as used in this article*, refers to the emergence of organ- and tissue-specific ageing trajectories within an individual, shaped by interactions between development, genome-exposome dynamics and evolutionary trade-offs. We value the use of this term to describe different patterns of ageing across tissues because it intuitively unifies previously used descriptors of uneven ageing under a common conceptual umbrella.

Evidence for the mosaic nature of ageing is accumulating across levels of biological organization. This relatively novel concept is supported by new analytical techniques such as high-resolution molecular atlases, often displaying distinct trajectories across body organs [11,21–24]. Whether organ-specific signatures predict disease risk, resilience or mortality remains one of the central challenges emerging from this view of ageing, providing a new framework for biomedicine and evolutionary biology. Ageing in one organ can influence others through circulatory, neural or inflammatory pathways, forming networks between the different systems [21,25]. Interventions that slow deterioration in one organ may ultimately help slow organismal ageing. Recognizing inter-organ connections and the underlying causes and mechanisms governing such processes can allow inferring systemic ageing and help to develop organ-targeted strategies [13].

From an evolutionary standpoint, mosaic ageing is particularly interesting when considering that natural selection targets traits and functions, rather than acting on organisms as indivisible units [26,27]. Organs differ in metabolic activity, growth or regenerative potential during a lifetime, including across life transitions [28–31]. These processes can potentially influence the organ and organismal vulnerability to stress and impact components of fitness such as reproductive success or lifespan. Consequently, it is plausible that ageing could evolve through differential selective pressures across functional body parts [32–35], depending on whether cross-organ connections are plastic or rigid [36]. Evolutionary theory predicts that the strength and timing of selection acting on different functions can generate heterogeneous ageing trajectories across organs, although the direction of these patterns may depend on how age-specific selection gradients operate on each trait [17,37,38].

In this article, we promote research on mosaic ageing that integrates insights from molecular biology, biomedicine and evolutionary ecophysiology. We first examine the mechanistic and functional basis of organ-specific ageing, highlighting how metabolic pathways and trade-offs can generate distinct ageing trajectories both at the local and systemic level (figure 1). We then evaluate classical and emerging tools that enable tracking organ-specific ageing, and discuss their challenges and limitations. Finally, we consider the implications of this approach for personalized medicine and evolutionary theory (figure 1). Ageing, we argue, may not be a single process but a layered architecture of decline and resilience that mirrors the modular design of living systems.

**Figure 1.**
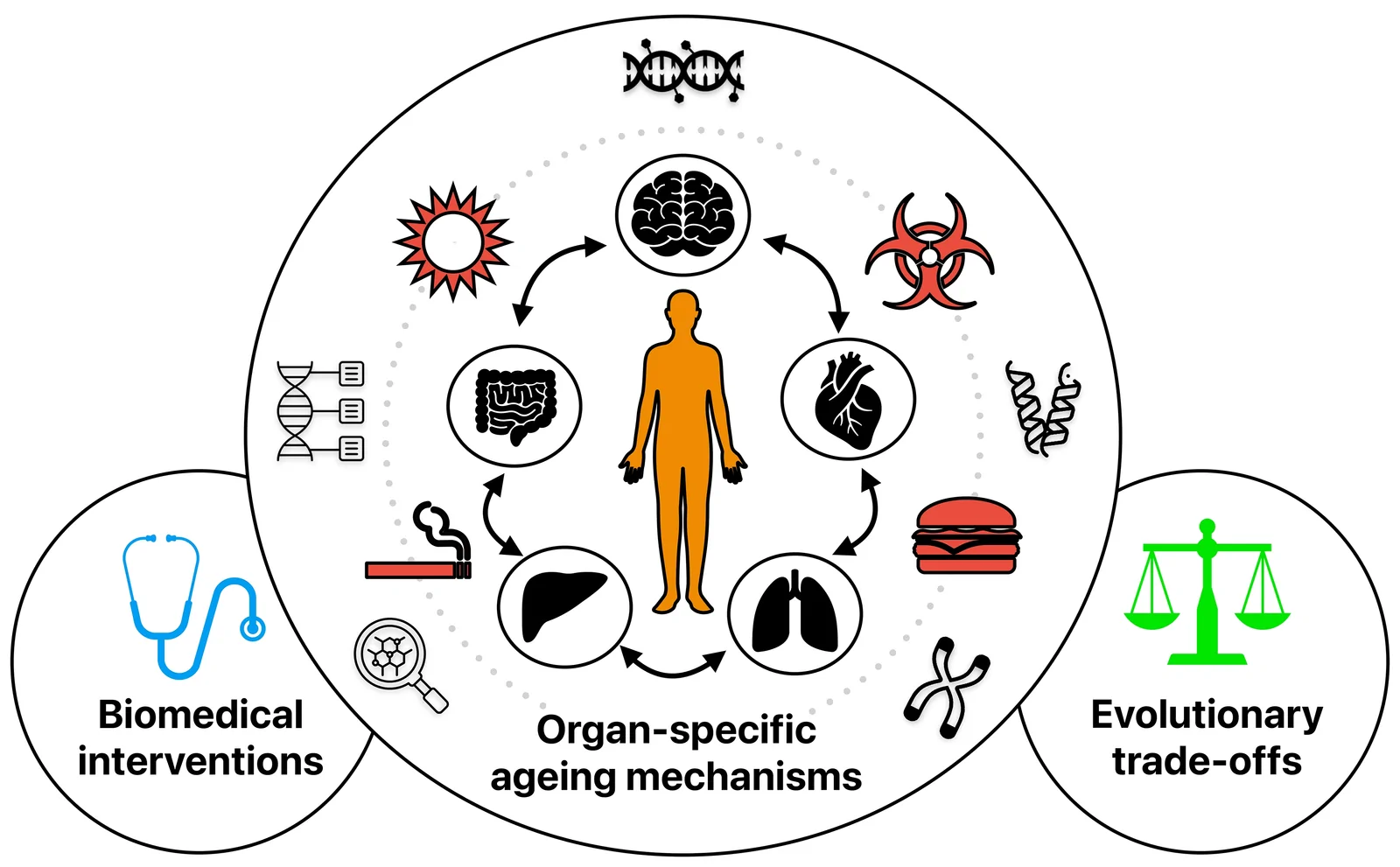
Perspective on integrating organ-specific ageing with proximate factors, biomedical interventions and evolutionary trade-offs. Organ-specific ageing mechanisms emerge from interactions between the genome, lifetime environmental exposure (the exposome) and developmental processes. These mechanisms can generate heterogeneous ageing trajectories across organs, which can influence disease risk, organismal resilience and fitness. At the medical level, this heterogeneity supports organ-targeted approaches to assess health span. At the evolutionary level, differences in organ-specific vulnerability reflect physiological trade-offs and may underpin population resilience to environmental stress as well as divergence under changing conditions.

## 2. The pieces of the puzzle: causes and consequences of organ-specific ageing

It has long been known that not all tissues age synchronously, as heterogeneity can be readily observed in morphological and behavioural traits such as greying hair, skin wrinkles and physical ability. As early as 2002, it was found that *Caenorhabditis elegans* shows differential patterns of senescence across tissue types [39], supporting the notion of a complex multivariate and multifaceted ageing process. At the time, the authors noted that part of the heterogeneity was owing to genetics and part seemed stochastic, which raised the question of the extent to which mosaic ageing is programmed [39]. Much progress has been made in this area through research focusing on the proximate mechanisms of organ- and tissue-specific ageing, and their contribution to whole-organism functional decline [11]. In this section, we advocate the importance of placing such mechanisms into the context of evolutionary theory (see box 2 for key concepts), encompassing both the proximate and ultimate dimensions. We subsequently outline conceptual links between organ-specific ageing and mortality, emphasizing the functional dimension.

**Box 2. Evolutionary concepts we use relevant to mosaic ageing**

Evolutionary biology has profoundly shaped contemporary ageing research, providing integrative frameworks to explain why senescence occurs and why it varies across environmental contexts [40]. Several concepts commonly used in the field are particularly relevant to mosaic ageing, in which tissues and organs can follow different trajectories of decline and differ in their consequences for organismal functioning. The definitions below summarize key evolutionary terms and principles referred to throughout this article.

**Fitness** refers to an individual's genetic contribution to future generations through survival and reproduction.

**Somatic maintenance, trade-offs**and **disposable soma theory**. **Somatic maintenance** refers to investment in preserving tissues other than the germline through repair and physiological homeostasis. **Trade-offs** refer to situations in which improvement in one function decreases another because resources are finite. Accordingly, increased investment in one function may reduce investment in others, creating trade-offs among maintenance, growth and reproduction. In this line, the **disposable soma theory** states that organisms allocate resources to reproduction and somatic maintenance, prioritizing reproductive success over indefinite survival [41]. Note that the assumption of environmental constraint limiting resources has proven difficult to demonstrate empirically; lab studies often show that reduced food intake prolongs longevity and reduces fecundity. Nevertheless, the theory has been highly influential, and experimental manipulations of reproductive effort have been shown to shape senescence in different taxa [42,43].

**Declining force of natural selection with age.** Natural selection refers to the differential survival and reproduction of individuals carrying heritable traits. Because fewer children are born from older parents, selection acts most strongly on traits affecting early-life survival and reproduction. Conversely, late-acting effects typically experience weaker selection, a pattern often termed the selection shadow [44]. This principle underlies classic evolutionary theories such as **mutation accumulation** [45], whereby deleterious late-acting mutations are imperfectly removed by selection, and **antagonistic pleiotropy** [46], in which genes or pathways that enhance early-life performance have detrimental effects later in life yet remain favoured by selection.

**Developmental quasi-programmes.** Proposed in modern ageing theory, quasi-programmes refer to biological processes that are beneficial during growth and development but continue into adulthood where they cause deleterious effects [47]. Their continued activity during adulthood may contribute to latent dysfunction, tissue damage and ageing. Since these negative effects occur during the late life phase, selection is weak and offset by the positive early life effects, in line with antagonistic pleiotropy.

### (a) Proximate and ultimate dimension

Evolutionary theory postulates that ageing evolves because the force of natural selection declines during the adult phase from the age of reproductive maturity [44]. Since selection is weaker at older ages, alleles with beneficial effects before reproductive maturity are favoured, even when those alleles incur some delayed cost [48]. In his seminal paper, Williams illustrated this with a hypothetical allele that increases calcium deposition, beneficial for bone development, but whose continued expression later in life would cause arteriosclerosis [46]. Subsequently, numerous alleles with antagonistic-pleiotropic effects have been widely reported [49]. This single-allele paradigm provides a useful starting point to understand the fundamental ecophysiology of mosaic patterns of ageing across tissues and organs when extended from one-to-many loci. Indeed, the physiological basis of mosaic ageing lies in the interplay between lifetime environmental exposures (exposome) and the genome (figure 1). The interfacing physiology is hierarchical (genes, proteins, cells, tissues and organs) and interactive, both horizontally and vertically, across levels of biological organization [50,51]. On the genetic level, differential gene expression occurs through external and internal factors influencing gene- and multi-omic regulatory networks. Although this complex physiology is not yet fully understood, key proximate components consist of non-coding RNAs, histone modification, acetylation, DNA methylation and chromatin remodelling [52,53], all of which are fundamentally implicated in early development [54,55]. Considering the evolutionary antagonistic pleiotropy theory of ageing, we can assume that natural selection prioritizes the optimization of this complex physiology for development until reproductive maturity. Seen through the lens of organ- or tissue-specific ageing, late-life sub-optimal gene expression with deleterious effects occurs as the developmental programme continues in the mechanistic sense, because factors that optimize late-life gene regulation rarely evolve in the absence of late-life selection pressures. This is referred to as the ‘quasi-programmatic’ theory of senescence [56,57], named such to indicate ageing is not programmed [47] (for a detailed discussion see [58]). One major challenge is to determine the causes of heterogeneity in tissue- and organ-specific ageing onset and trajectory, and whether they can be generalized. Given that heterogeneity in organ-specific ageing trajectories results from the interactions between the static genome that is inherited, lifestyle and environmental conditions and mutations [59], many causes of heterogeneity are likely to be specific to individuals. However, some aspects of tissue-specific ageing could be more common, such as atherosclerosis, which is clearly linked to both lifestyle and genetics, in the sense that the combination of certain gene variants and lifestyle factors almost invariably leads to this disease [60]. Unravelling the exposome-genome interplay with sufficient resolution will be crucially important for progressing our understanding of the mechanisms of ageing and the potential for biomedical interventions that may prolong health span.

A recent study using transcriptome data from multiple organs and tissues supports the theory of quasi-programmed senescence and heterogeneity, highlighting how this type of research is of crucial importance [61]. The study shows that tissue-specific expression follows a pattern of early-life divergence up to sexual maturity, followed by late-life convergence in certain tissues, potentially associated with loss of tissue and cell identity [61]. Early-life divergence in tissue-specific gene expression is a feature of developmental processes governing cell differentiation and tissue specialization. During this developmental process, most cells in our body form identity and become canalized in their function. Subsequent convergence in tissue-specific gene expression could suggest the (partial) reversal, a process during which tissues lose their identity, possibly reverting to an ancestral state after which tissue and organ functions deteriorate [62]. The developmental dimension could be particularly important because developmental processes can leave long-lasting signatures on organ resilience. This perspective has recently been extended to neurodegeneration, where early neurodevelopmental trajectories may modulate vulnerability to late-life brain decline [63]. Tissue-specific gene expression patterns exhibiting divergence and convergence provide a molecular explanation for antagonistic pleiotropic effects in a slightly different way than quasi-programmed senescence as outlined above. Although technically challenging, multi-tissue transcriptome analyses offer a powerful approach for identifying causal interactions among organs that contribute to ageing-related disease. In addition to transcriptomic data, it will also be important to continue to investigate the underlying causal factors that underpin tissue and organ-specific gene expression patterns with age. DNA methylation clocks provide meaningful clues to which genes might matter most in which specific environmental context, as many DNA methylation changes with age appear to be organ- and tissue-specific [64]. Some of these topics are discussed further in §4.

### (b) Functional biomedical dimension

Conceptually, mosaic ageing can manifest in divergent or convergent patterns among organs with variation in the onset and rate of ageing. Many of the qualitative and quantitative aspects (i.e. which tissues, when and how fast they age) are likely highly specific to the individual and their lifetime exposure to the environment. As outlined above, targeted molecular (epi)genomic approaches could shed light on the proximate mechanisms underpinning ageing heterogeneity among organs and may also serve as biomarker tools for tracking these processes [23,50,51]. Ultimately, the development of this field has important implications for biomedical applications aimed at assessing health and ageing-related disease risk as well as targeted interventions. Among others, exosomes are potentially important in this context. Exosomes are endosomal extracellular vesicles that carry important resources including proteins, lipids, mRNAs and non-coding RNAs. They are key mediators of intercellular communication and implicated in senescence [65]. Recent studies identified a potential role for bioengineered exosomes in targeted therapeutic interventions owing to their efficiency and specificity [66]. Although there are many hurdles on the road still, this is but one example of how measurements of organ-specific state, lifestyle and genomic factors, and bioengineering could be integrated to deliver targeted interventions that improve healthspan.

In the simplest conceptual scenario of mosaic ageing, organs show a uniform onset of ageing with linear trajectories but variation in their rate of ageing and baseline hazard rate. In the example shown in figure 2, brain, liver and heart have similar rates of ageing across both scenarios (left versus right panel in figure), but their rankings in baseline mortality are reversed. This leads to divergence of hazard rates (figure 2A) and convergence (figure 2B), which has important implications for the link between organ failure and organism mortality. In both scenarios, death will occur when one of the three organs reaches the threshold for lethality (indicated by the horizontal dashed line). This conceptual view of multi-level ageing centres on the idea that failure of one among different physiological sub-units can trigger organismal collapse. Such a threshold model was considered unlikely by Maynard Smith, who argued that natural selection should favour synchronous senescence [67]. However, here we argue that because the force of natural selection declines at older ages, it is unlikely that selection consistently optimizes the degree of synchrony among organs. Rather, we deem it more likely that mosaic ageing results from the genome–exposome interactions specific to individuals, and that many of these heterogeneous trajectories have only limited fitness consequences because they arise at ages when the force of natural selection has already declined.

**Figure 2.**
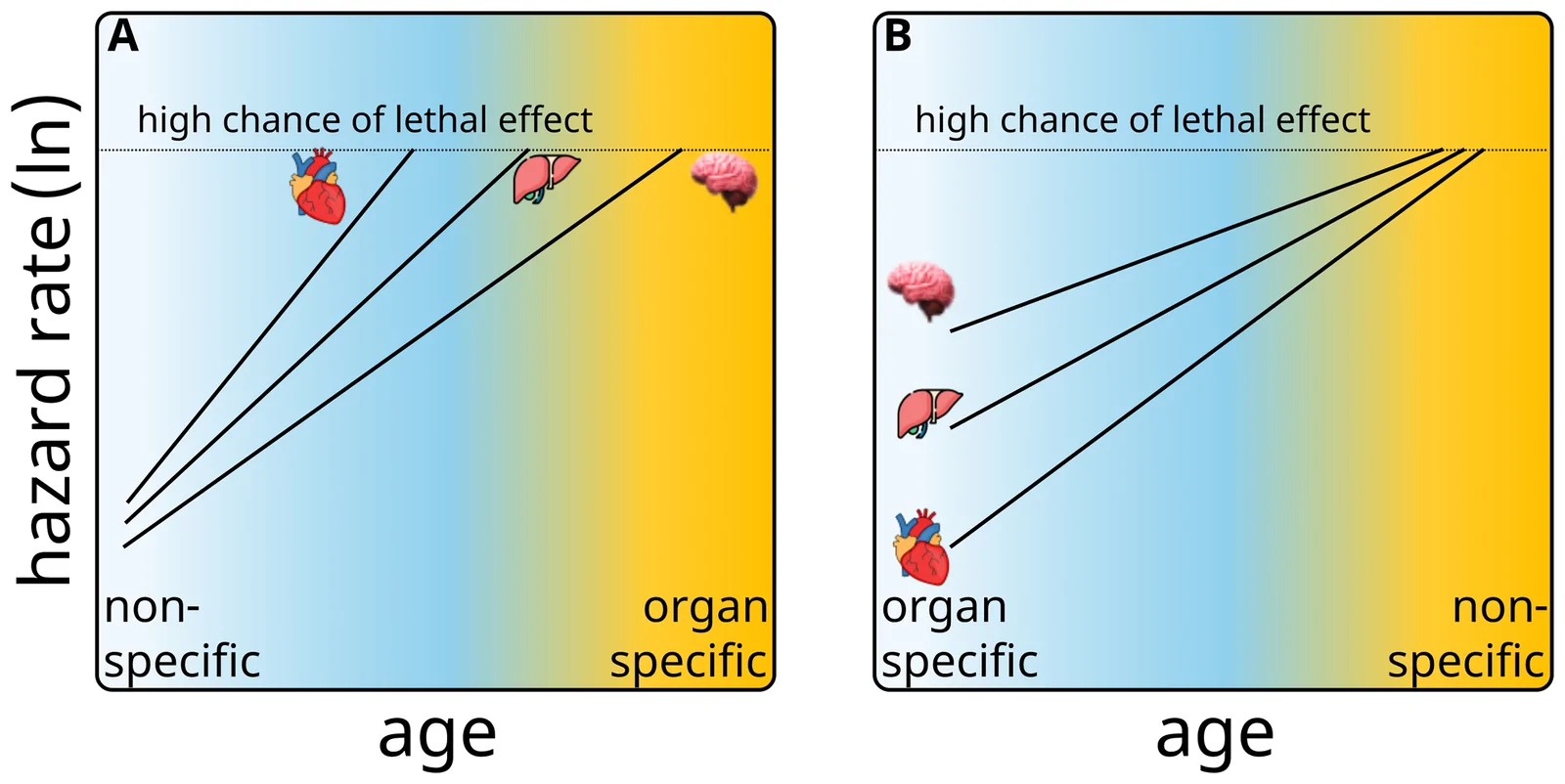
Divergent and convergent patterns of organ-specific ageing trajectories. The colour gradient reflects organism mortality risk from low (light blue) to high (deep orange). The linear lines reflect how the natural logarithm of the hazard rate of individual organs increases with age. Organ failure causes mortality when its trajectory reaches the threshold ‘high chance of lethal effect’. (A) Organs show a uniform starting point of senescence but vary in their rate of functional deterioration. This most likely triggers mortality by failure of the organ most susceptible to ageing. In this scenario, targeted medical intervention delaying or reversing ageing of the weakest organ could increase healthspan. (B) Organs show different levels of robustness (redundancy) and vary in their rate of functional deterioration as predicted by redundancy models, resulting in convergence. In this scenario, the cause of death becomes less organ-specific with increasing age, limiting opportunity for increasing healthspan through medical intervention.

The notion of a multi-subsystem ‘bauplan’ consisting of parallel units that can trigger system failure is well founded in systems reliability theory [68], and when applied to biological systems can (at least conceptually) explain individual variation in the cause of death. From this theoretical background, we can make some novel predictions for biological systems. The example shown in figure 2B illustrates that the cause of death becomes increasingly unpredictable with age because the organ failure trajectories converge and reach the threshold of lethality within a narrow temporal window. Convergence is an emergent property of a system layout in which organs show different levels of redundancy after development is completed, and in which redundancy units show a uniform rate of failure [69]. In this example, one could consider cells as redundancy units, with each cell exhibiting the same average failure rate within and across organ tissues. The organ with larger initial redundancy is more vulnerable to damage as a greater number of redundancy elements are at risk initially, but this difference diminishes over time as the number of elements at risk narrows across organs [69]. Determining if organs show divergent or convergent patterns of deterioration will be paramount for predicting the cause of death and developing intervention strategies [70].

In figure 2, we used linear ageing trajectories in this simple schematic scenario, merely to illustrate the point that our ability to use organ-specific ageing data to predict health risks depends on the pattern of convergence or divergence. However, the magnitude of differences among age-specific organ states depends on the specific organ ageing trajectories, which may be nonlinear and even show reversals after successful interventions. Finally, the latest research suggests that organs’ state may influence the ageing of other organs (e.g. [13,71]), suggesting there may exist a dynamic network of interlinked systems. This will most likely confound predictions about the cause of death because inter-organ interactions can lead to unpredictable outcomes. Much more theoretical and empirical research is needed in this area. We advocate the heuristic value of systems reliability theory and other network-based modelling approaches [72] to further our understanding of inter-organ dynamics. From a computational perspective, this is now within reach with the advent of AI and machine learning-based approaches [73]. Alongside these conceptual and computational advances, classical and emerging biomarkers have provided key empirical insights into organ-specific ageing and its contribution to predicting mortality.

## 3. Telomere dynamics: a classical window into mosaic ageing

Numerous molecular pathways have historically been proposed as universal ageing biomarkers [9,74–76]; however, they often vary strongly with shifts in organ metabolism and environmental conditions. For instance, oxidative stress markers have provided important insights into ageing and physiological condition across biomedical and ecological systems [77–79], although their repeatability and associations with being fitness-related often vary among biomarkers, tissues, life stages, taxa and environmental contexts [76,80–84]. Among the classical candidates, telomere biology stands out as one of the earliest, most widely adopted and conceptually influential molecular marker approaches to quantify biological ageing within populations and across species [85–87].

Telomeres are repetitive DNA sequences accompanied by protein complexes located at linear chromosome ends that typically shorten with cell division and cumulative physiological stress [88]. Telomere length is predictive of mortality in humans and non-model organisms, and telomere attrition is considered a hallmark of ageing [74,89,90]. A substantial body of work shows that telomere dynamics vary markedly across tissues within individuals, reinforcing the concept of organ-specific ageing. On a conceptual level, these organ-specific telomere dynamics could reflect differences in somatic redundancy across tissues [70], i.e. variation in the capacity of organs to cope with damage while maintaining their functionality. This may lead to convergent ageing patterns later in life, as depicted in figure 2 and specifically predicted by redundancy models. In addition to differences in telomere length, early comparative analyses in humans revealed heterogeneous shortening rates among organs, with proliferative tissues such as liver and kidney showing clear age-related attrition, whereas largely post-mitotic tissues such as cerebral cortex and myocardium exhibit little or no decline [91]. Later human studies confirmed strong cross-tissue correlations in telomere length despite persistent organ-specific differences, suggesting that much of the between-tissue variation is established early in life and maintained into adulthood [92,93]. Together, this paints a rather complex picture, raising important questions about the causes and consequences of tissue-specific telomere dynamics, difficult to address in human studies.

Evidence from non-human vertebrates mirrors this pattern. In birds and reptiles, telomere length across organs is often positively correlated, although the strength and direction of these relationships depend on sex and life-history context [94–96]. Developmental and energetic processes can further decouple organ-specific telomere dynamics. For example, in tree swallows, growth-maintenance relationships differ among organs during early life, with organ-specific associations between growth rate and telomere length emerging in brain and adrenal cells but not in liver or blood [97]. Experimental activation of telomerase, the enzyme responsible for telomere maintenance, seems to produce divergent responses among organs, highlighting limits to uniform telomere protection across the body [98]. Environmental stressors can amplify this heterogeneity [99]. Chronic radiation exposure in wild mammals, for instance, alters telomere length and telomerase expression in an organ-specific manner, weakening within-individual correlations [100]. Consistent with this view, recent work in amphibians demonstrates that metamorphic remodelling, environmentally driven shifts in growth and developmental trajectories, and early thermal conditions can generate long-lasting effects on telomere dynamics, including organ-dependent trajectories that persist into adulthood [101–103]. Yet telomere variation across organs is not universally reshaped by life-history strategy. For instance, in the Japanese quail, individuals selected for divergent reproductive investment show strong cross-organ correlations despite absolute differences [104]. In addition, most of these patterns are likely shaped in part by broad differences in metabolic strategy, as contrasting thermal physiology strategies (i.e. ectotherms versus endotherms) may influence the degree of cross-organ coupling [105–108].

Despite their widespread use and conceptual appeal, telomere-based metrics present important limitations. Individual variation in telomere length is to a large extent driven by heritable factors and only partially covaries with longevity, and this heritable component appears to be largely maintained across the lifespan [109–114], although whether its magnitude differs among organs remains unclear. This suggests that telomeres may in part reflect intrinsic organismal quality rather than purely cumulative damage [115], while their causal contribution to lifespan variation is unclear [116]. Despite telomere dynamics clearly capturing important aspects of within-body ageing heterogeneity, their context-dependence and modest links to survival highlight the limitations of relying on telomere length and attrition as single biomarkers for characterizing the multi-tissue complexity of ageing. These limitations indicate the need for higher-resolution, integrative approaches capable of resolving organ-specific ageing dynamics across biological scales.

## 4. Emerging high-resolution tools to track mosaic ageing

Recent technological advances have greatly improved our ability to capture the complexity of biological ageing across tissues and levels of biological organization. Among these approaches, the study of the methylome and the development of epigenetic clocks have likely been the most influential [117,118]. This approach is based on the idea that DNA methylation, most commonly at cytosine-guanine dinucleotides (CpG sites), changes in predictable ways as an organism ages [118]. Although DNA methylation also occurs at non-CpG sites and involves different modified bases and oxidation states, most epigenetic clocks are based on CpG methylation patterns. Machine learning models use DNA methylation patterns to estimate an individual's ‘epigenetic age’ through the development of epigenetic clocks [119,120]. These clocks can predict chronological age with remarkable accuracy, with correlation coefficients typically above 0.90 in many species; this means an average error of 2–4 years and two to three weeks in humans and mice, respectively [121–123]. Intriguingly, in humans, changes in DNA methylation profiles compared to healthy individuals have been observed to be linked to age-related diseases such as hypertension, Alzheimer's or different types of cancer [23,124]. Also, interventions promoting longevity and reducing age-related diseases, such as calorie restriction, are capable of shifting the methylome to a ‘younger profile’ by altering the DNA methylation profiles in several tissues [121,122,125]. Current research aims to develop tissue-specific or even single-cell clocks to capture the ageing patterns specific to tissues or cell types [126–128]. Others go further, combining DNA methylation with gene expression or protein data into multi-omic ageing clocks [51]. However, the use of epigenetic clocks still has several challenges ahead. First, methylation profiles often show high variation across tissues and cells, and can be strongly influenced by environmental conditions, complicating the development of pan-tissue epigenetic clocks that accurately predict biological age across many different tissues [23]. Most clocks have been trained on human and mouse blood samples, which limits their generalization to other tissues or species. Epigenetic clocks show strong correlations with chronological age; hence their causal connection to the processes governing biological ageing remains limited. From an eco-evolutionary perspective, epigenetic clocks can greatly improve our knowledge about how environmental stress, life-history trade-offs and phenotypic plasticity shape ageing in natural populations [129,130]. Nonetheless, their application in wild systems remains challenging owing to the highly context-dependent nature of epigenetic ageing and requires the integration of molecular, ecological and demographic data. Recent work has begun to extend epigenetic clock approaches to non-model organisms, including bats, cetaceans and elephants, highlighting their potential in ecological and evolutionary contexts [122,131,132]. By contrast, for many species that are challenging to study in the wild, such as small insects, it remains unclear if epigenetic clocks can be successfully implemented [129].

Like studies of the methylome, the use of transcriptomics (genome-wide gene expression), metabolomics (cellular metabolic profiles) and proteomics (large-scale protein expression) has advanced our understanding of mosaic ageing [133,134]. Transcriptomics have revealed broad age-related shifts in gene expression, including upregulation of inflammatory pathways, impaired mitochondrial function and a general loss of transcriptional regulation [135–137]. The magnitude and direction of these changes often differ markedly among tissues, consistent with the divergent and convergent organ-specific patterns of ageing [11]. Metabolomics and proteomics provide another layer of insight, as they capture functional changes and can predict health outcomes. Metabolomic research linked to ageing is still scarce, but it has already demonstrated, for instance, the central role of components related to energy metabolism and redox balance in ageing, often found to be tissue-specific [133,138]. Large-scale proteomic analyses, such as those conducted in human plasma, have quantified thousands of proteins (>3000) in thousands of individuals (>4000) to estimate a ‘proteomic age’, showing nonlinear alterations in the plasma proteome, with waves of changes in the fourth, seventh and eighth decades of human life [139]. Other studies have linked changes in the proteomic profile to the probability to die, finding, for example, that each extra year of proteomic age relative to chronological age was associated with a 13% higher hazard of mortality [140]. In the context of mosaic ageing, for instance, tissue-specific transcriptomic profiling in ageing mouse liver and kidney further revealed that mRNA and protein levels are often decoupled, highlighting post-transcriptional and post-translational regulation as major contributors to changes in the proteome during ageing [141,142]. Proteomic heterogeneity also emerges across tissues and cell types, as distinct organs can show unique proteomic ageing signatures; for example, in the brain, recent work has identified cell-type- and region-specific shifts in protein phosphorylation [143]. Finally, integrating single-cell technologies into *omics* approaches is providing evidence for the mosaic nature of ageing at the microscale, revealing substantial heterogeneity within tissues, with some cells maintaining stable molecular identities while others undergo pronounced age-related drift [144].

Despite offering a more comprehensive view of ageing, these technologies face substantial challenges as they often require large, high-quality datasets and sophisticated computational pipelines, limiting their application to model organisms under controlled conditions. Integrating data across organs and time remains technically difficult, and distinguishing true biological signals from noise is a persistent difficulty. Biogerontological research has identified candidate pathways for rejuvenation and tissue repair; however, their application across eco-evolutionary contexts (where ageing must be understood across species, environments and life histories) remains virtually unknown.

## 5. Moving towards causal inference

While an expanding array of molecular markers now captures age-associated patterns across tissues and cells, it is still often unclear whether these markers reflect mechanistic drivers of functional decline or merely correlate with the process of ageing. Establishing which molecular changes causally contribute to age-related organ dysfunction, loss of resilience and reduced survival, rather than serving as passive readouts of ageing, has emerged as a central challenge in contemporary ageing research [145].

Tissue-specific longitudinal (i.e. within-individual) data remain rare in studies investigating organ-specific ageing trajectories, likely because repeated sampling is often methodologically challenging and highly invasive or destructive. However, minimally invasive biopsy techniques such as image-guided biopsies [146], together with improved tissue and single-cell extraction protocols, are beginning to enable repeated sampling in accessible organs such as skin, muscle or liver, offering the possibility to track molecular changes within individuals. Although still limited, these approaches may provide invaluable data for calibrating and validating functional biomarkers, and to link their dynamics to disease risk, physiological resilience or life-history trade-offs. Likewise, imaging techniques such as positron emission tomography (PET) or magnetic resonance imaging (MRI) can capture organ-specific decline in real time through non-invasive longitudinal observation [147,148]. Combining longitudinal functional imaging with cross-sectional molecular measurements can improve our knowledge of ageing across different levels of complexity, from molecular damage and organ performance to organismal survival.

Despite this increased level of resolution, causality in ageing studies often remains elusive. Targeted experimental interventions can shed light on this issue. For instance, pharmacological treatments and genetic or epigenetic manipulations, including CRISPR-based approaches and/or RNAi, allow selective perturbation of pathways related to ageing in specific tissues. *In vitro* systems may further enable bottom-up dissection of some of these mechanisms under controlled conditions [149]. Combining these approaches with longitudinal phenotyping could allow testing whether physiological pathways such as telomere dynamics or redox changes causally drive differential ageing among organs. At the analytical level, causal inference approaches such as Mendelian randomization and target-trial emulation could help disentangle whether specific physiological pathways causally drive differential ageing among organs [150–152]. Mendelian randomization uses genetic variants as natural experiments to test whether variation in a molecular trait leads to functional decline (e.g. [153,154]), whereas target-trial emulation leverages large longitudinal or observational datasets to mimic experimental manipulation (e.g. [155]).

Experimental evolution provides an additional powerful framework for achieving causal inference in ageing research, as it enables the manipulation of selective pressures on lifespan, reproduction and environmental stressors across generations. By tracking coordinated responses across molecular, physiological and life-history traits, experimental evolution can help disentangle whether candidate biomarkers of ageing are true drivers of senescence or merely correlated signatures [156], and it is a powerful approach that may be used to unveil cryptic trade-offs [157]. At a time when high-resolution and longitudinal data are increasingly accessible, there is a unique opportunity to integrate these layers into a coherent framework that explains why ageing unfolds unevenly across organs and contexts, and how this mosaic shapes health, resilience and functional and physiological trade-offs.

## 6. Future research avenues and conclusions

One big question remains: how malleable or rigid mosaic ageing trajectories are. As previously mentioned, ageing of one organ may accelerate ageing of another. For instance, Tian *et al.* [13] showed that ageing of the pulmonary system leads to faster cardiovascular ageing, which in turn results in faster ageing of the musculoskeletal and renal systems. This suggests a process not unlike the tumbling of domino blocks leading to ever-cascading effects. But is this a universal pattern that applies to other tissues? Is it broadly consistent across environmental contexts, individuals and species? Can the domino blocks be repositioned to intervene and circumvent negative cascading effects? In other words, are the connections underpinning multivariate ageing hardwired in our physiology or genetics, or are they malleable by the environment, implying some scope for biomedical interventions? A deep dive into the mosaics of ageing could shed light on these important questions. If resources and tissue access were unlimited, future research could integrate bottom-up molecular measurements across multiple tissues with top-down organismal approaches, ideally in wild animal systems. This would enable mapping how natural conditions interact with specific physiological subunits and tissues, as well as estimating their fitness correlations. When such approaches are combined with granular quantitative genomics scaled to the population, such studies could reveal crucial cross-tissue negative genetic correlations, i.e. the direction the domino block falls, as well as individual variation. This would constitute an important step towards understanding the detailed genetic and molecular basis of mosaic ageing, its universality across organs, tissues and individuals, and its implications for health span and for interventions aimed at unlinking the domino blocks.

The evidence synthesized here shows that ageing is best described as a mosaic cross-tissue process, in which organs follow distinct trajectories shaped by development, exposome, lifestyle and evolutionary constraints. Accordingly, organism-wide averages or single biomarkers cannot fully capture ageing dynamics, as decline and resilience are unevenly distributed across tissues. This perspective instead points to ageing as an emergent property of interacting biological sub-systems, under selection to function optimally during early adult life, but inevitably leading to heterogeneous tissue-specific declines at old age. Bottom-up organ-specific and multi-omic approaches provide the resolution needed to track within-individual heterogeneity, but their value ultimately depends on linking molecular patterns to function, causality and survival. Achieving this integration will require closer crosstalk between molecular and cell biologists, evolutionary ecologists and ecophysiologists. Ultimately, adopting a mosaic framework shifts the focus from describing ageing patterns to explaining their mechanistic origins and may help resolve one of biology's most persistent enigmas.

## Data Availability

This article has no additional data.

## Theme

One contribution of 21 to a theme issue ‘Developmental circuits in ageing’.
